# Naïve CD8^+^ T-Cells Engage a Versatile Metabolic Program Upon Activation in Humans and Differ Energetically From Memory CD8^+^ T-Cells

**DOI:** 10.3389/fimmu.2018.02736

**Published:** 2018-12-21

**Authors:** Francesco Nicoli, Laura Papagno, Justin J. Frere, Mariela Pires Cabral-Piccin, Emmanuel Clave, Emma Gostick, Antoine Toubert, David A. Price, Antonella Caputo, Victor Appay

**Affiliations:** ^1^INSERM, Centre d'Immunologie et des Maladies Infectieuses, Sorbonne Université, Paris, France; ^2^Department of Molecular Medicine, University of Padua, Padua, Italy; ^3^Department of Immunobiology and the Arizona Center on Aging, University of Arizona College of Medicine Tucson, Tucson, AZ, United States; ^4^Institut Universitaire d'Hématologie, Université Paris Diderot, Sorbonne Paris Cité, Paris, France; ^5^INSERM UMR 1160, Laboratoire d'Immunologie et d'Histocompatibilité, Hôpital Saint-Louis, AP-HP, Paris, France; ^6^Division of Infection and Immunity, Cardiff University School of Medicine, Cardiff, United Kingdom; ^7^International Research Center of Medical Sciences, Kumamoto University, Kumamoto, Japan

**Keywords:** immunometabolism, mTOR, naïve T-cells, priming, CD8^+^ T-lymphocytes

## Abstract

**Background:** Characterization of the intracellular biochemical processes that regulate the generation and maintenance of effector and memory CD8^+^ T-cells from naïve precursors is essential for our understanding of adaptive immune responses and the development of immunotherapies. However, the metabolic determinants of antigen-driven activation and differentiation remain poorly defined, especially in humans.

**Methods:** We used a variety of different approaches, including gene expression profiling and measurements of nutrient flux, to characterize the basal and activation-induced energetic requirements of naïve and phenotypically-defined subsets of human memory CD8^+^ T-cells.

**Findings:** Profound metabolic differences were apparent as a function of differentiation status, both at rest and in response to stimulation via the T cell receptor (TCR). Of particular note, resting naïve CD8^+^ T cells were largely quiescent, but rapidly upregulated diverse energetic pathways after ligation of surface-expressed TCRs. Moreover, autophagy and the mechanistic target of rapamycin (mTOR)-dependent glycolytic pathway were identified as critical mediators of antigen-driven priming in the naïve CD8^+^ T cell pool, the efficiency of which was dampened by the presence of neutral lipids and fatty acids.

**Interpretation:** These observations provide a metabolic roadmap of the CD8^+^ T-cell compartment in humans and reveal potentially selective targets for novel immunotherapies.

## Introduction

CD8^+^ T-cells play a key role in the adaptive immune system, enabling the recognition and elimination of intracellular pathogens and various cancers (1). Protective immunity in this lymphocyte compartment originates from antigen-driven priming events, which trigger the activation and differentiation of naïve precursors, seeding qualitatively diverse populations of memory CD8^+^ T-cells. The kinetics of expansion and the acquisition of effector functions within the emergent antigen-experienced pool can also be manipulated using targeted interventions to beneficial effect (2–5). However, our knowledge of the intracellular biochemical processes that govern the behavior of human lymphocytes as a function of lineage and differentiation status remains incomplete. It is established that naïve CD8^+^ T-cells undergo a metabolic transition in response to activation, switching from a primary reliance on mitochondrial respiration to a primary reliance on aerobic glycolysis (6–8). *In vivo* mouse studies have further shown that the bioenergetics of CD8^+^ T-cell activation vary as a function of antigen exposure (9), suggesting that metabolic reprogramming is regulated across the differentiation spectrum via cognate engagement of surface-expressed T-cell receptors (TCRs). To consolidate this paradigm, especially in light of current efforts to augment immune efficacy using nutrient-based strategies (10, 11), it is necessary to extend these studies into humans (8, 12–14).

In this study, we investigated the basal and activation-induced energetic requirements of naïve and memory CD8^+^ T-cells, aiming to create a metabolic roadmap spanning the lymphocyte differentiation spectrum in humans (15). Considerable metabolic heterogeneity was observed among phenotypically-defined subsets of human CD8^+^ T-cells. Moreover, autophagy and mechanistic target of rapamycin (mTOR)-induced glycolysis cooperatively regulated the expansion and functionality of antigen-specific CD8^+^ T-cells, and TCR-induced activation was influenced by neutral lipids and fatty acids (FAs).

## Materials and Methods

### Human Subjects and Samples

This study was approved by the Comité de Protection des Personnes of the Pitié Salpétrière Hospital (Paris). All participants provided written informed consent in accordance with the Declaration of Helsinki. Venous blood samples were collected from 41 healthy volunteers (median age 39 years, age range 19–65 years, 56% females). Peripheral blood mononuclear cells (PBMCs) were isolated from acid citrate dextrose collection tubes via density gradient centrifugation according to standard protocols and cryopreserved in complete medium supplemented with 10% dimethyl sulfoxide and 20% fetal calf serum (FCS). Complete medium (R+) consisted of RPMI 1640 supplemented with non-essential amino acids, penicillin-streptomycin (100 U/mL), L-glutamine (2 mM), and sodium pyruvate (1 mM).

### Flow Cytometry and Cell Sorting

PBMCs were surface stained in the dark for 15 min at room temperature with directly conjugated monoclonal antibodies. αCD3, αCD4, αCD8, αCD27, αCD45RA, αCD49d, αCD57, and αCCR7 were used to identify different CD8^+^ T-cell subsets (Figure S1; Table S1). Non-viable cells were eliminated from the analysis using LIVE/DEAD Fixable Aqua (Life Technologies). Activation status was assessed using αCD38, αCD40L, αCD69, αCD134, αHLA-DR, and αPD-1. In priming assays, cells were stained first in the dark with PE-conjugated ELA/HLA-A2 tetramers for 15 min at 37°C. Intracellular staining for granzyme B and Tbet was performed using a Transcription Factor Buffer Set (BD Pharmingen). Samples were acquired using a Fortessa flow cytometer (BD Biosciences). CD8^+^ T-cell subsets were sorted using a FACSAria II flow cytometer (BD Biosciences). Data were analyzed using FACSDiva version 7.0 (BD Biosciences) and FlowJo version 10 (Tree Star Inc.).

### RNA Extraction, Retrotranscription, and qPCR Analysis

PBMCs were activated for 5 h with plate-bound αCD3, stained as described above, and sorted at 300 cells/subset directly into lysis buffer (Macherey-Nagel). After RNA extraction and cDNA synthesis, specific targets were amplified using PreAmp Master Mix (Fluidigm). Gene expression profiling was conducted using a Biomark (Fluidigm) with EvaGreen Supermix (Bio-Rad). Relative levels of each RNA species were calculated using the 2^−ΔΔCT^ method with reference to a housekeeping gene (human 18S). Heatmaps were constructed using Omics Explorer software (Qlucore).

### Metabolic Profiling by Flow Cytometry

To determine glucose uptake, neutral lipid content, or FA uptake, PBMCs were incubated in PBS with 50 μM 2′-(N-(7-nitrobenz-2-oxa-1,3-diazol-4-yl)amino)-2-deoxyglucose (2-NBDG), 10 μM 4,4-difluoro-1,3,5,7,8-pentamethyl-4-bora-3a,4a-diaza-*s*-indacene (BODIPY™ 493/503), or 1μM 4,4-difluoro-5,7-dimethyl-4-bora-3a,4a-diaza-*s*-indacene-3-hexadecanoic acid (BODIPY™ FL C16), respectively, for 20 min at 37°C (all reagents from Thermo Fisher Scientific). To determine cholesterol uptake or mitochondrial mass, PBMCs were incubated in R+ with 22-(N-(7-nitrobenz-2-oxa-1,3-diazol-4-yl)amino)-23,24-bisnor-5-cholen-3β-ol(NDB-cholesterol) as per the manufacturer's instructions (Cayman Chemical) or with 500 nM Mitotracker Deep Red (Thermo Fisher Scientific), respectively, for 30 min at 37°C. To determine the production of reactive oxygen species (ROS), PBMCs were incubated in R+ with 5 μM CellROX Green Reagent (Thermo Fisher Scientific) for 15 min at room temperature. To determine mitochondrial membrane potential, PBMCs were incubated in R+ with 25 nM tetramethylrhodamine, methyl ester, perchlorate (TMRM, Thermo Fisher Scientific) for 30 min at 37°C. To determine autophagic activity, PBMCs were stained using a CYTO-ID Autophagy Detection Kit as per the manufacturer's instructions (Enzo Life Sciences) for 30 min at 37°C. To determine mTOR activity, PBMCs were incubated in BD Cytofix Fixation Buffer (BD Biosciences) for 10 min at 37°C, washed, incubated in BD Phosflow Perm Buffer III (BD Biosciences) for 30 min on ice, washed again, and stained for phospho-S6 ribosomal protein (Ser235/236, Cell Signaling Technology) for 1 h at room temperature. Additional stains were used as described above to characterize the metabolic profile of distinct CD8^+^ T-cell subsets in each assay.

### Assessment of Metabolic Pathways Involved in T-Cell Activation

PBMCs were incubated overnight with the following compounds to inhibit specific metabolic pathways: glycolysis, 5 nM 2-deoxy-D-glucose (2-DG, Sigma-Aldrich); glutaminolysis, 10 μM bis-2-(5-phenylacetamido-1,3,4-thiadiazol-2-yl)ethyl sulfide (BPTES, Sigma-Aldrich); mTOR, 5 nM rapamycin (Sigma-Aldrich); autophagy, 10 μM spautin-1 (a kind gift from Dr. Stephanie Graff-Dubois); FA oxidation, 100 μM etomoxir (Sigma-Aldrich); FA synthesis, 25 μM irgasan (Sigma-Aldrich); and cholesterol synthesis, 1 μM simvastatin (Sigma-Aldrich). Pre-treated cells were cultured under resting conditions or activated for 24 h with plate-bound αCD3, then surface stained as described above to measure the expression of activation markers by flow cytometry. The activation/inhibition ratio was measured for each T-cell subset using the following formula: 1 – (% HLA-DR^+^ on activated cells with inhibitors – % HLA-DR^+^ on resting cells)/(% HLA-DR^+^ on activated cells – % HLA-DR^+^ on resting cells). Spanning-tree progression analysis of density-normalized events (SPADE) was conducted using three activation markers (CD134, HLA-DR, and PD-1), and t-distributed stochastic neighbor embedding (t-SNE) was used to check the clustering generated via SPADE.

### *In vitro* Priming of Antigen-Specific CD8^+^ T-Cell Precursors

Naïve precursors specific for the HLA-A2-restricted epitope ELAGIGILTV (ELA) were primed *in vitro* as described previously (16, 17). Briefly, thawed PBMCs were resuspended in AIM medium (Invitrogen), plated at 2.5 × 10^6^ cells/well in a 48-well tissue culture plate in the absence or presence of different metabolic inhibitors, and stimulated with the peptide YTAAEELAGIGILTVILGVL, which contains the optimal epitope in heteroclitic form, at a concentration of 1 μM together with FLT3 ligand (50 ng/mL, R&D Systems). After 24 h (day 1), maturation was induced via the addition of TLR8L (0.5 μg/mL), L-carnitine (20 μM), or a cytokine cocktail incorporating TNF-α (1,000 U/mL), IL-1β (10 ng/mL), IL-7 (0.5 ng/mL), and PGE2 (1 μM) (all reagents from R&D Systems). On day 2, the medium was supplemented at a volume ratio of 10% with FCS (Gibco). On days 5 and 8, the medium was replaced with fresh RPMI 1640 containing 10% FCS (Gibco). The frequency and phenotype of ELA-specific CD8^+^ T-cells were determined on day 10.

## Results

### Resting Naïve and Memory CD8^+^ T-Cells Exhibit Distinct Energetic Requirements

It has been established that resting and activated T-cells rely on different biochemical and signal-transduction pathways (8, 18). However, it is less clear if such differences also exist among resting subpopulations at various stages of differentiation. To address this issue, we analyzed the expression profile of a selection of genes in naïve and distinct subsets of memory CD8^+^ T-cells (Figure S1).

**Figure 1:**
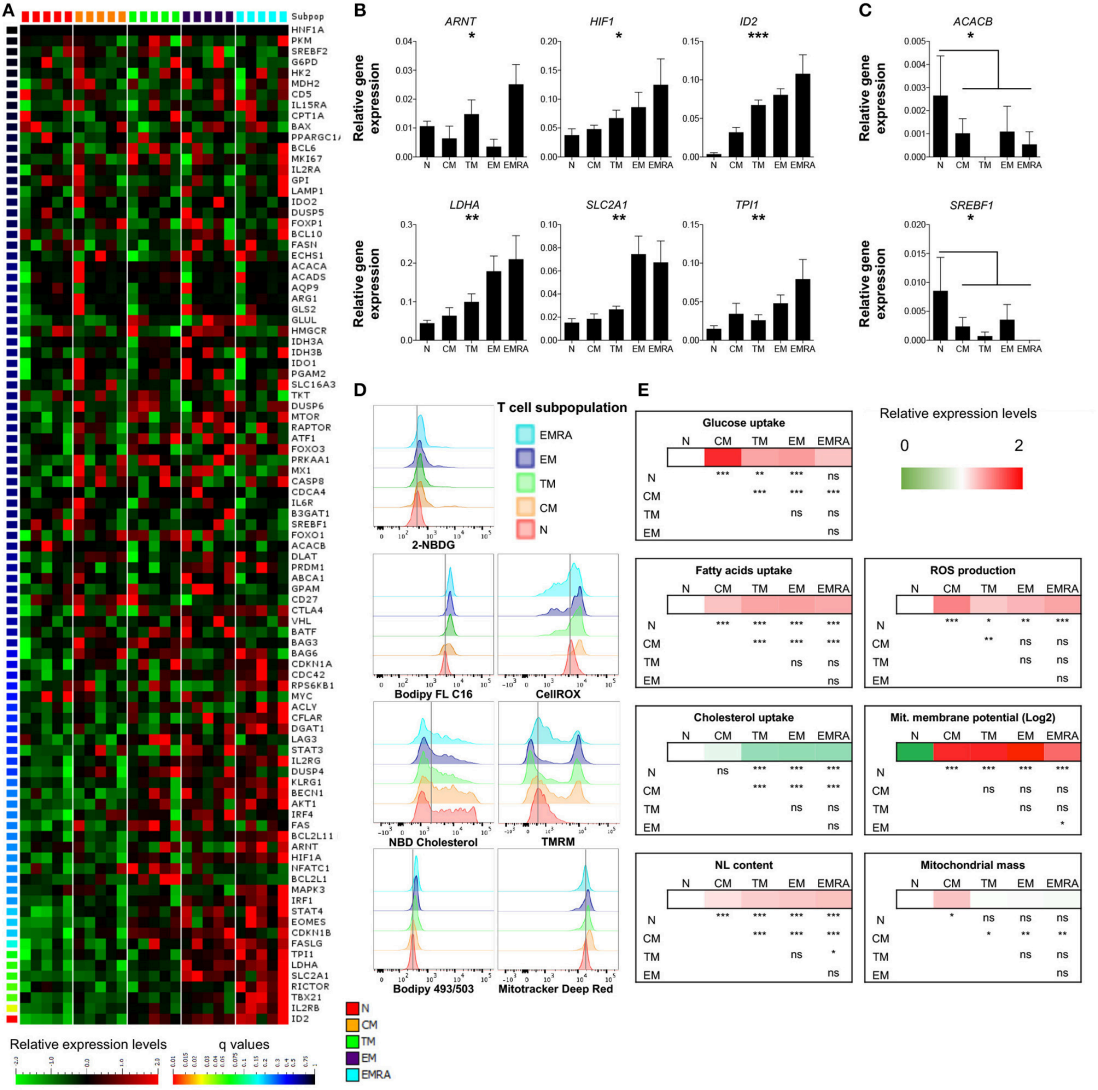
Basal energetic requirements of distinct CD8^+^ T-cell subsets. **(A)** Heatmap showing the gene expression profile (according to *q*-value) of resting CD8^+^ T-cell subsets. N (CCR7^+^, CD45RA^+^, CD27^+^); CM (CCR7^+^, CD45RA^−^, CD27^+^); TM (CCR7^−^, CD45RA^−^, CD27^+^); EM (CCR7^−^, CD45RA^−^, CD27^−^); EMRA (CCR7^−^, CD45RA^+^, CD27^−^). *N* = 5. **(B,C)** Relative mean expression of metabolism-related genes with significant differences among T-cell subsets **(B)** or in comparisons of N vs. the whole memory compartment **(C)**. *N* = 5. **(D,E)** Representative examples **(D)** and heatmaps showing relative mean expression levels **(E)** of various metabolic properties measured by flow cytometry (glucose uptake with 2-NBDG; FA uptake with Bodipy FL C16; cholesterol uptake with NDB cholesterol; neutral lipid (NL) content with Bodipy 493/503; ROS production with CellROX; mitochondrial membrane potential with TMRM; mitochondrial mass with Mitotracker Deep Red). *N* = 10. Statistical significance was determined using a one-way paired ANOVA with Bonferroni's post-test **(A–C)** or a paired Student's *t*-test **(E)**. *P < 0.05, **P < 0.01, ***P < 0.001; ns, not significant.

As expected, several genes associated with differentiation were poorly expressed in naïve CD8^+^ T-cells and highly expressed in effector memory (EM) and terminally differentiated effector memory (EMRA) CD8^+^ T-cells. Differentially regulated products included transcription factors (Eomes, Tbet, Stat4, and IRF1), intracellular signaling molecules (Rictor), Fas ligand, and the IL-2 receptor (Figure 1A; Table S2). Notably, five genes involved in glucose metabolism (*ARNT*, aryl hydrocarbon receptor nuclear translocator, also known as hypoxia-inducible factor HIF-1β; *HIF1A*, HIF-1α; *LDHA*, lactate dehydrogenase A; *SLC2A1*, solute carrier family 2 member 1, also known as GLUT1; and *TPI1*, triosephosphate isomerase 1) and the transcription factor *ID2* (inhibitor of DNA binding 2), which controls lipid metabolism (19), were expressed at progressively higher levels along the T-cell differentiation pathway (Figure 1B; Table S2). Naïve CD8^+^ T-cells also expressed higher levels of *ACACB* (acetyl-CoA carboxylase-β) and *SREBF1* (sterol regulatory element binding transcription factor 1), which are involved in lipid biosynthesis and cholesterol transport, compared with memory CD8^+^ T-cells (Figure 1C; Table S2). These data confirmed previous observations in mice showing that metabolism is controlled as a function of differentiation within the CD8^+^ T-cell lineage (20). An irreversible program of genetically regulated metabolic changes therefore accompanies the transition from quiescent naïve to antigen-experienced memory status in the human adaptive immune system.

To extend these observations, we assessed the metabolic properties of different CD8^+^ T-cell subsets by measuring the uptake and storage of various nutrients, as well as mitochondrial functions and the production of ROS. Glucose uptake was minimal among resting CD8^+^ T-cells, especially within the naïve compartment (Figures 1D,E; Table S3). Naïve CD8^+^ T-cells also displayed lower levels of neutral lipids and FA uptake compared with memory CD8^+^ T-cells (Figures 1D,E; Table S3). In contrast, cholesterol uptake was higher among naïve CD8^+^ T-cells compared with transitional memory (TM), EM, and EMRA CD8^+^ T-cells, but not significantly different compared with central memory (CM) CD8^+^ T-cells (Figures 1D,E; Table S3). Mitochondrial membrane potential was very low among naïve CD8^+^ T-cells, which likewise produced small amounts of ROS relative to memory CD8^+^ T-cells (Figures 1D,E; Table S3).

Among the different memory subsets, CM CD8^+^ T-cells were notably distinct from TM, EM, and EMRA CD8^+^ T-cells, which exhibited broadly similar metabolic properties (Figures 1D,E; Table S3). In particular, glucose and cholesterol uptake were significantly higher, mitochondrial mass was significantly greater, and FA uptake was significantly lower among CM CD8^+^ T-cells compared with other resting memory CD8^+^ T-cells (Figures 1D,E; Table S3). Higher levels of ROS production were also observed among CM CD8^+^ T-cells compared with naïve and TM CD8^+^ T-cells (Figures 1D,E; Table S3).

Collectively, these findings suggested that: (i) naïve CD8^+^ T-cells exist in a low energy state, based on minimal nutrient uptake and mitochondrial activity; and (ii) memory CD8^+^ T-cells exhibit metabolic variability across the differentiation spectrum.

### Activation of Naïve CD8^+^ T-Cells Triggers a Rapid Metabolic Switch

**Figure 2:**
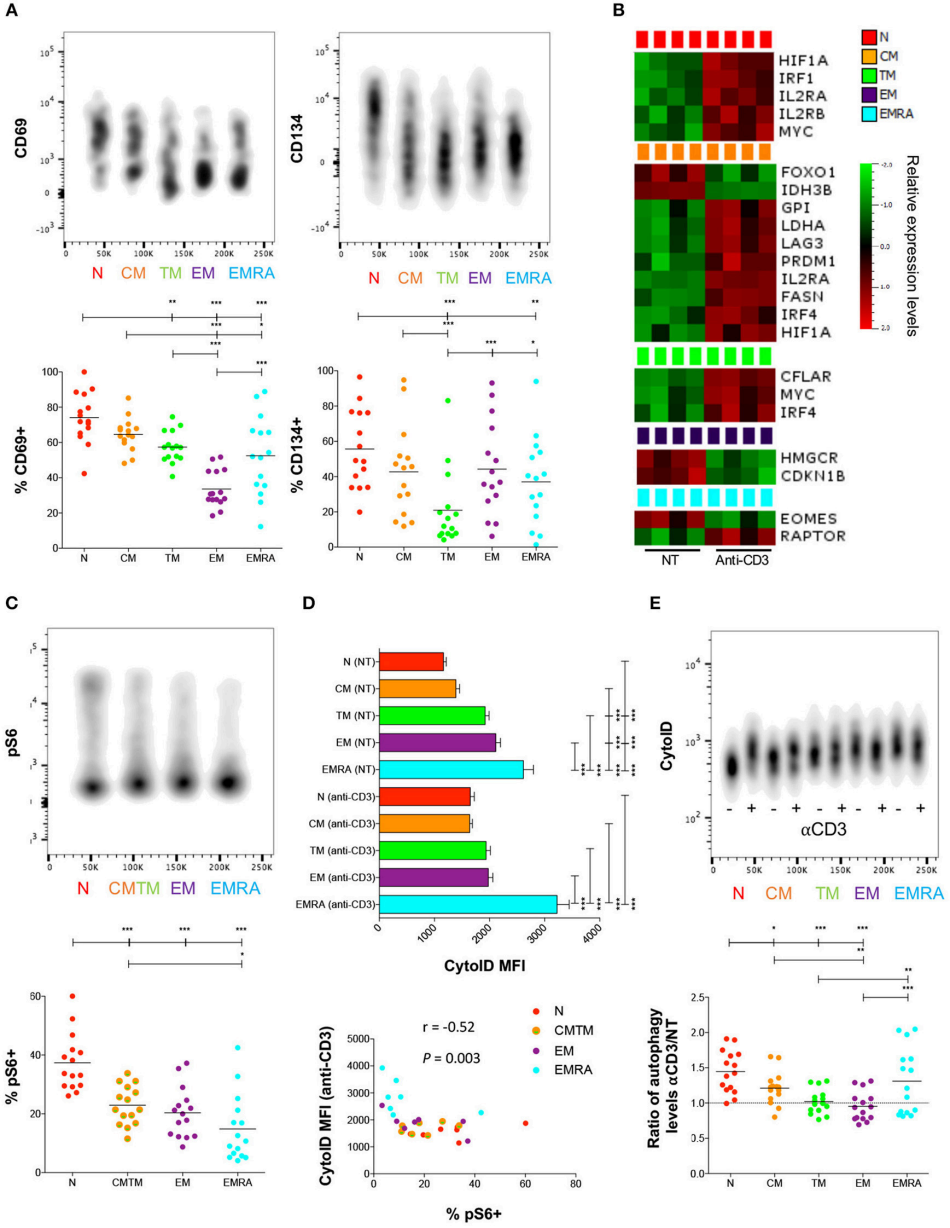
Metabolic switch in activated naïve CD8^+^ T-cells. **(A)** PBMCs were activated with plate-bound αCD3. Expression of CD69 and CD134 was measured by flow cytometry after 3 h and 24 h, respectively. Upper panels: one representative example is shown for each stain. Lower panels: horizontal lines depict mean values. *N* = 15. **(B)** Heatmap showing significant differences in gene expression between resting (NT) and αCD3-activated CD8^+^ T-cells after 3 h. *N* = 4. **(C)** PBMCs were activated with plate-bound αCD3. Expression of pS6 was measured by flow cytometry after 3 h. Upper panel: one representative example is shown. Lower panel: horizontal lines depict mean values. *N* = 15. **(D)** PBMCs were activated with plate-bound αCD3. Autophagic activity was measured by flow cytometry after 24 h. Upper panel: autophagic activity in resting (NT) or αCD3-activated CD8^+^ T-cells. Error bars depict mean ± SEM. Lower panel: correlation between autophagy and mTOR activity upon activation. *N* = 15. **(E)** PBMCs were activated with plate-bound αCD3. Activation-induced autophagic activity is shown as a ratio (αCD3/NT). Upper panel: one representative example is shown. Lower panel: horizontal lines depict mean values. *N* = 15. Statistical significance was determined using a one-way paired ANOVA with Bonferroni's post-test **(A,C–E)**, a paired Student's *t*-test **(B)**, or Spearman's rank correlation **(D)**. *P < 0.05, **P < 0.01, ***P < 0.001.

To investigate the link between antigen-driven priming events and basal metabolic requirements, we stimulated PBMCs via generic ligation of TCRs and measured the upregulation of early (CD69 after 3 h) and intermediate activation markers (CD134 after 24 h). Naïve CD8^+^ T-cells expressed higher levels of these activation markers at the corresponding time points compared with memory CD8^+^ T-cells (Figure 2A; Table S4). In addition, PD-1 was strongly upregulated on the surface of naïve CD8^+^ T-cells in response to activation (Figure S2; Table S4). This rapid phenotypic transformation was associated with markedly increased expression of several genes (Figure 2B), including *IL2RA* and *IL2RB*, as well as the mTOR-induced transcription factors *HIF1A* and *IRF1* (interferon regulatory factor 1), which support a metabolic switch to glycolysis (21), and *MYC*, which plays a key role in activation-induced metabolic reprogramming across multiple pathways (22). These data confirmed previous studies (21, 23) suggesting that naïve CD8^+^ T-cell activation triggers a metabolic switch regulated via mTOR.

On this basis, we quantified the phosphorylated form of ribosomal protein S6 (pS6), a key downstream target of mTOR. Naïve CD8^+^ T-cells contained significantly higher levels of pS6 compared with memory CD8^+^ T-cells (Figure 2C; Table S5). In addition, we measured autophagic activity, which is commonly downregulated in association with activation of the mTOR pathway (24). Naïve CD8^+^ T-cells displayed significantly lower levels of autophagic activity compared with highly differentiated memory CD8^+^ T-cells (Figure 2D). Activation-induced autophagic activity further correlated inversely with pS6 levels across all CD8^+^ T-cell subsets (Figure 2D). Nonetheless, autophagic activity was markedly upregulated in naïve CD8^+^ T-cells after stimulation, whereas less pronounced activation-induced shifts were observed in CM, TM, and EM CD8^+^ T-cells (Figure 2E; Table S5).

Among the different memory subsets, CM CD8^+^ T-cells responded more vigorously to stimulation compared with TM, EM, and EMRA CD8^+^ T-cells, most notably at the early time point (Figure 2A), and upregulated several genes associated with activation and differentiation (Figure 2B), including *IL2RA, LAG3*, and the transcription factors *IRF4* and *PRDM1* (Blimp-1) (25). These changes were linked with increased levels of autophagy and mTOR activity (Figure 2C; Table S5), which mirrored the expression of activation markers induced via TCR-mediated signals (Figure 2A). Activated CM CD8^+^ T-cells also upregulated genes involved in glycolysis, such as *GPI* (glucose-6-phosphate isomerase), *LDHA*, and *HIF1A*, and FA synthesis (*FASN*, fatty acid synthase) (Figure 2B). In contrast, activated EM and EMRA CD8^+^ T-cells exhibited minimal changes in gene expression and limited upregulation of mTOR, in line with the findings of a recent study (12).

Collectively, these findings suggested that resting naïve CD8^+^ T-cells and, to a lesser extent, resting CM CD8^+^ T-cells, rapidly upregulate diverse metabolic pathways in response to activation signals transduced via surface-expressed TCRs.

### Activation of Naïve CD8^+^ T-Cells Relies on Autophagy and Glycolysis

**Figure 3:**
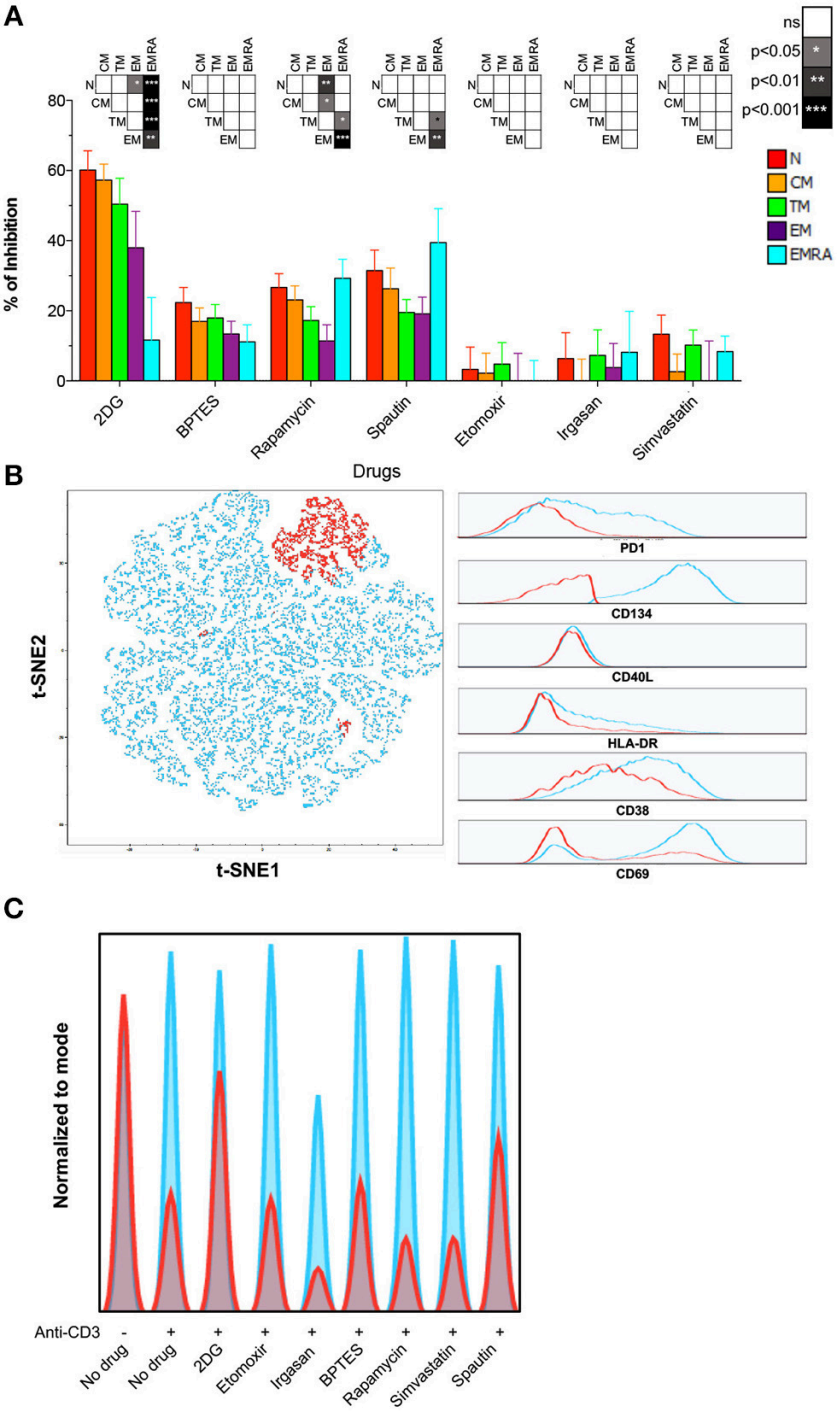
Role of autophagy and glycolysis in naïve CD8^+^ T-cell activation. **(A)** PBMCs were incubated for 24 h with different metabolic inhibitors and activated with plate-bound αCD3. Inhibition of HLA-DR upregulation was measured by flow cytometry after 24 h. Bars depict mean ± SEM. Statistical significance was determined using a one-way paired ANOVA with Bonferroni's post-test. *N* = 10. **(B)** PBMCs were activated as in **(A)**. Expression of CD38, CD40L, CD69, CD134, HLA-DR, and PD-1 was measured after 24 h. Left panel: a *t*-SNE depiction of SPADE is shown for one representative sample. Right panel: expression of each marker is shown for the two clusters defined by SPADE. Highly activated (HA) CD8^+^ T-cells are shown in blue; poorly activated (PA) CD8^+^ T-cells are shown in red. **(C)** Histogram plots showing the proportion of HA (blue) and PA (red) among naïve CD8^+^ T-cells activated in the presence of different metabolic inhibitors (data from one representative donor).

To determine which metabolic pathways are necessary for the activation of naïve and memory CD8^+^ T-cells, we treated PBMCs with various inhibitors prior to stimulation and monitored subsequent upregulation of the activation marker HLA-DR. Inhibition of glycolysis with 2-DG dramatically impacted CD8^+^ T-cell activation as an inverse function of differentiation (Figure 3A; Table S6). Naïve CD8^+^ T-cells were inhibited to the greatest extent. Partially differentiated memory CD8^+^ T-cells were also more susceptible to 2-DG than EMRA CD8^+^ T-cells, consistent with the findings of a recent study (12). CM CD8^+^ T-cells were likewise inhibited to a comparable extent after blockade of glutaminolysis with BPTES or autophagy with spautin-1, indicating the concurrent use of different metabolic pathways (Figure 3A; Table S6).

To confirm these results, we measured activation using five additional markers (CD38, CD40L, CD69, CD134, and PD-1) 24 h after ligation of surface-expressed TCRs. Flow cytometric data were analyzed using an unsupervised approach, namely a combination of SPADE and t-SNE. This strategy allowed the identification of highly activated (HA) and poorly activated (PA) cells in clusters based on the composite expression of CD38, CD40L, CD69, CD134, HLA-DR, and PD-1 (Figure 3B). The effect of each inhibitor was then assessed using the HA/PA ratio (Figure 3C). Inhibition of autophagy and glycolysis again showed the most dramatic effect on naïve CD8^+^ T-cell activation (Figure 3C). In contrast, inhibition of the mevalonate pathway with simvastatin or inhibition of FA oxidation or synthesis with etomoxir or irgasan, respectively, did not prevent naïve CD8^+^ T-cell activation (Figure 3C).

Collectively, these findings demonstrated that naïve CD8^+^ T-cells rely primarily on autophagy and glycolysis for activation, whereas memory CD8^+^ T-cells display more complex and plastic metabolic requirements in response to functional engagement of surface-expressed TCRs.

### Antigen-Specific Priming of Naïve CD8^+^ T-Cells Depends on Autophagy and mTOR

**Figure 4:**
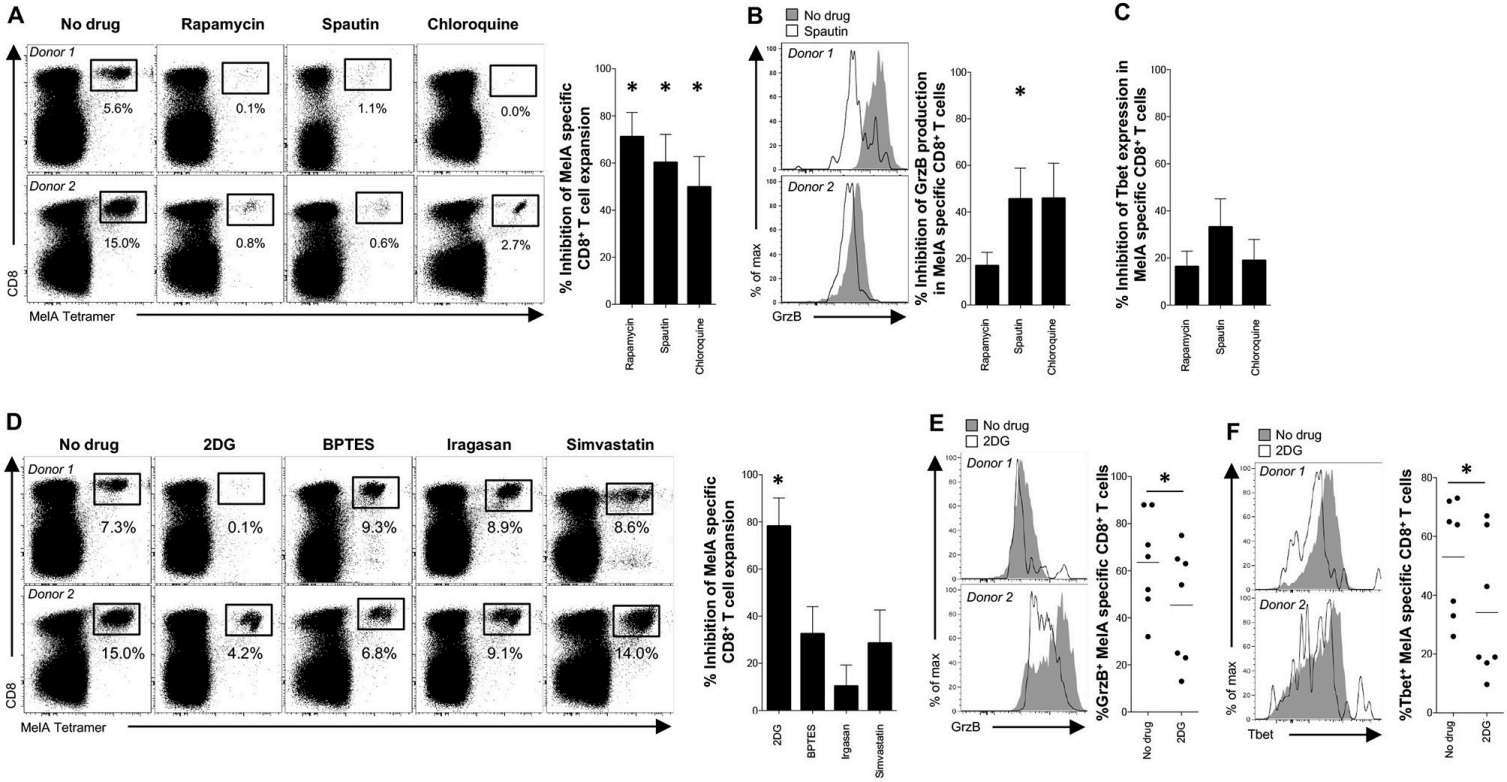
Engagement of autophagy and mTOR during antigen-specific priming of naïve CD8^+^ T-cells. **(A–F)** MelA-specific naïve CD8^+^ T-cells were primed in the absence or presence of various metabolic inhibitors. **(A,D)** MelA-specific CD8^+^ T-cells were quantified by flow cytometry after 10 days using cognate PE-conjugated ELA/HLA-A2 tetramers. Left panels: representative data are shown as dot plots. Right panels: percent inhibition of expansion is shown as mean ± SEM. **(B,E)** Expression of granzyme B in primed MelA-specific CD8^+^ T-cells was measured by flow cytometry after 10 days. Left panels: representative data are shown as histogram plots. Right panel **(B)**: percent inhibition of granzyme B expression is shown as mean ± SEM. Right panel **(E)**: percent expression of granzyme B is shown. Horizontal lines depict mean values. **(C,F)** Expression of Tbet in primed MelA-specific CD8^+^ T-cells was measured by flow cytometry after 10 days. **(C)** Percent inhibition of Tbet expression is shown as mean ± SEM. Left panel **(F)**: representative data are shown as histogram plots. Right panel **(F)**: percent expression of Tbet is shown. Horizontal lines depict mean values. Statistical significance was determined using the Wilcoxon signed rank test **(A–F)**. *N* = 7 **(A–C)**; *N* = 8 **(D)**; *N* = 7 **(E,F)**. *P < 0.05.

To probe the biological relevance of these findings, we conducted *in vitro* priming experiments with the model antigen Melan-A (MelA) (17). Inhibition of mTOR with rapamycin dramatically impaired the expansion of MelA-specific CD8^+^ T-cells (Figures 4A, S3A). Analogous effects were observed with spautin-1 and chloroquine, both of which block autophagic activity (Figure 4A). Moreover, granzyme B production was significantly inhibited in cultures pre-treated with spautin-1, but not in cultures pre-treated with rapamycin (Figures 4B, S3B). A comparable trend was observed in parallel analyses of Tbet expression (Figures 4C, S3C). These data suggested that antigen-driven priming of naïve CD8^+^ T-cell precursors depends on autophagy and the activity of mTOR.

Several activation-induced metabolic pathways, including glycolysis, glutaminolysis, and lipid synthesis, are known to be regulated by mTOR (26). In line with a key role for glycolysis, we found that pre-treatment with 2-DG, but not BPTES, irgasan, or simvastatin, markedly inhibited the *in vitro* expansion of MelA-specific CD8^+^ T-cells (Figures 4D, S3D). MelA-specific CD8^+^ T-cells primed in the presence of 2-DG also displayed low levels of granzyme B production and Tbet expression relative to MelA-specific CD8^+^ T-cells primed in the absence of inhibitors (Figures 4E,F). These results indicated that mTOR regulates naïve CD8^+^ T-cell priming via the glycolysis pathway (6–8).

### Fatty Acid Oxidation Enhances Effector Functions in Primed CD8^+^ T-Cells

**Figure 5:**
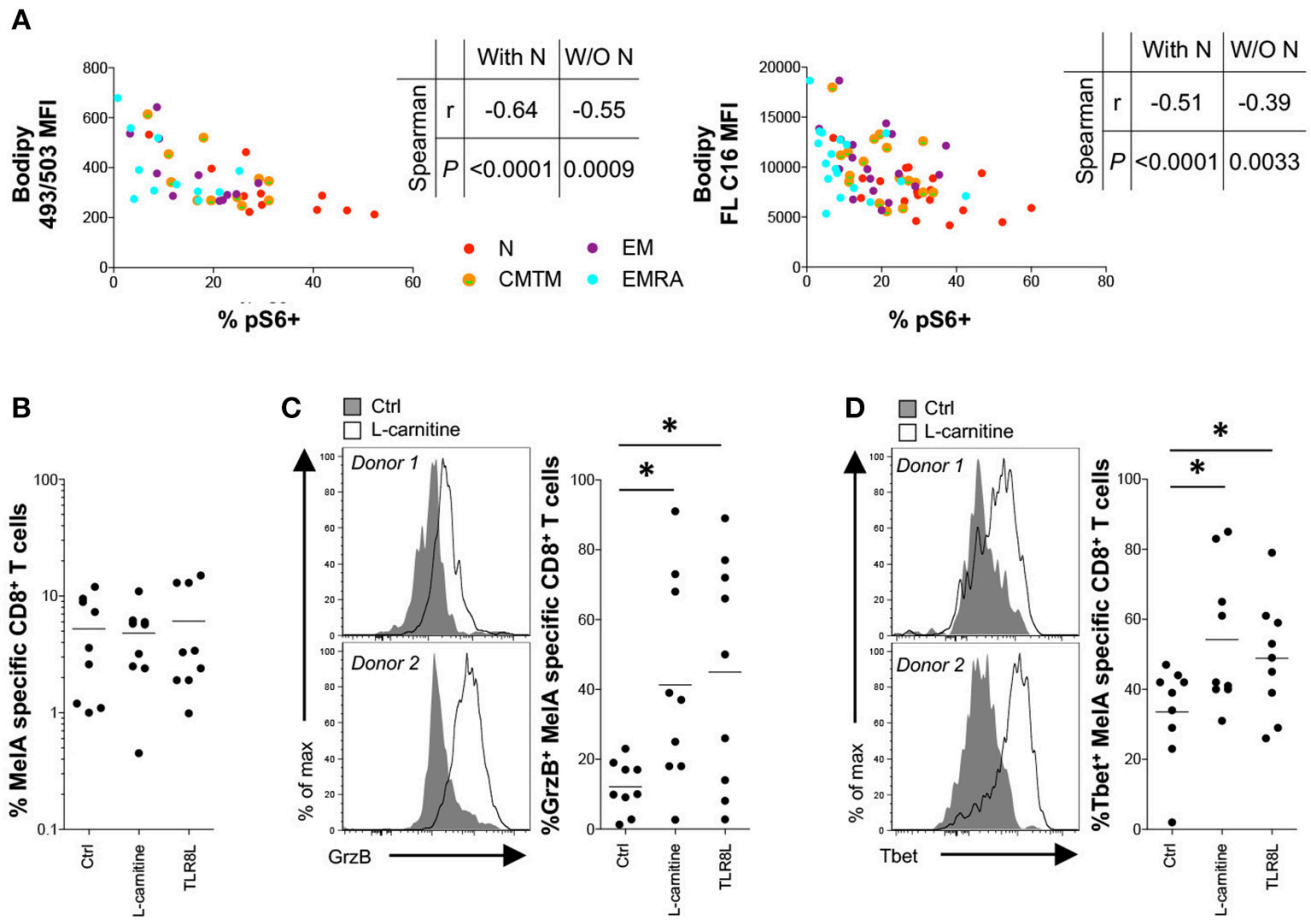
Effect of L-carnitine on the functionality of primed antigen-specific CD8^+^ T-cells. **(A)** Correlation between pS6 expression after activation for 3 h with plate-bound αCD3 and basal neutral lipid (NL) content (left panel) or fatty acid (FA) uptake (right panel). **(B–D)** MelA-specific naïve CD8^+^ T-cells were primed in the absence or presence of L-carnitine or TLR8L. MelA-specific CD8^+^ T-cells were quantified by flow cytometry after 10 days using cognate PE-conjugated ELA/HLA-A2 tetramers **(B)**. Expression of granzyme B **(C)** and Tbet **(D)** in primed MelA-specific CD8^+^ T-cells was measured by flow cytometry after 10 days. Left panels: representative data are shown as histogram plots. Right panels: percent expression of granzyme B or Tbet is shown. Horizontal lines depict mean values. *N* = 9. Statistical significance was determined using Spearman's rank correlation **(A)** or the Wilcoxon signed rank test **(B–D)**. *P < 0.05.

To synthesize these findings, we correlated basal energetic parameters with activation-induced mTOR activity. Neutral lipid content and FA uptake in the resting state correlated inversely with mTOR activity, even when naïve CD8^+^ T-cells were excluded from the analysis (Figure 5A). This observation was reminiscent of a previous study, which linked high concentrations of neutral lipids and FAs with altered lymphocyte fitness (27).

To assess the functional implications of this observation, we conducted *in vitro* priming experiments in the presence of L-carnitine, which promotes FA transport into the mitochondrial matrix. Pharmacological enhancement of fatty acid oxidation (FAO) has been shown previously to improve tumor-specific CD8^+^ T-cell reactivity in mice (28–30). Although L-carnitine exerted no detectable effects on the expansion of MelA-specific CD8^+^ T-cells (Figure 5B), it significantly increased granzyme B production and Tbet expression in primed MelA-specific CD8^+^ T-cells (Figures 5C,D). Of note, these mTOR-dependent molecules were upregulated in the presence of L-carnitine to levels observed in the presence of TLR8L, a potent adjuvant that enhances the *de novo* generation of effector CD8^+^ T-cells (17).

Collectively, these findings suggested that neutral lipids and FAs can impede the activation of mTOR.

## Discussion

**Figure 6:**
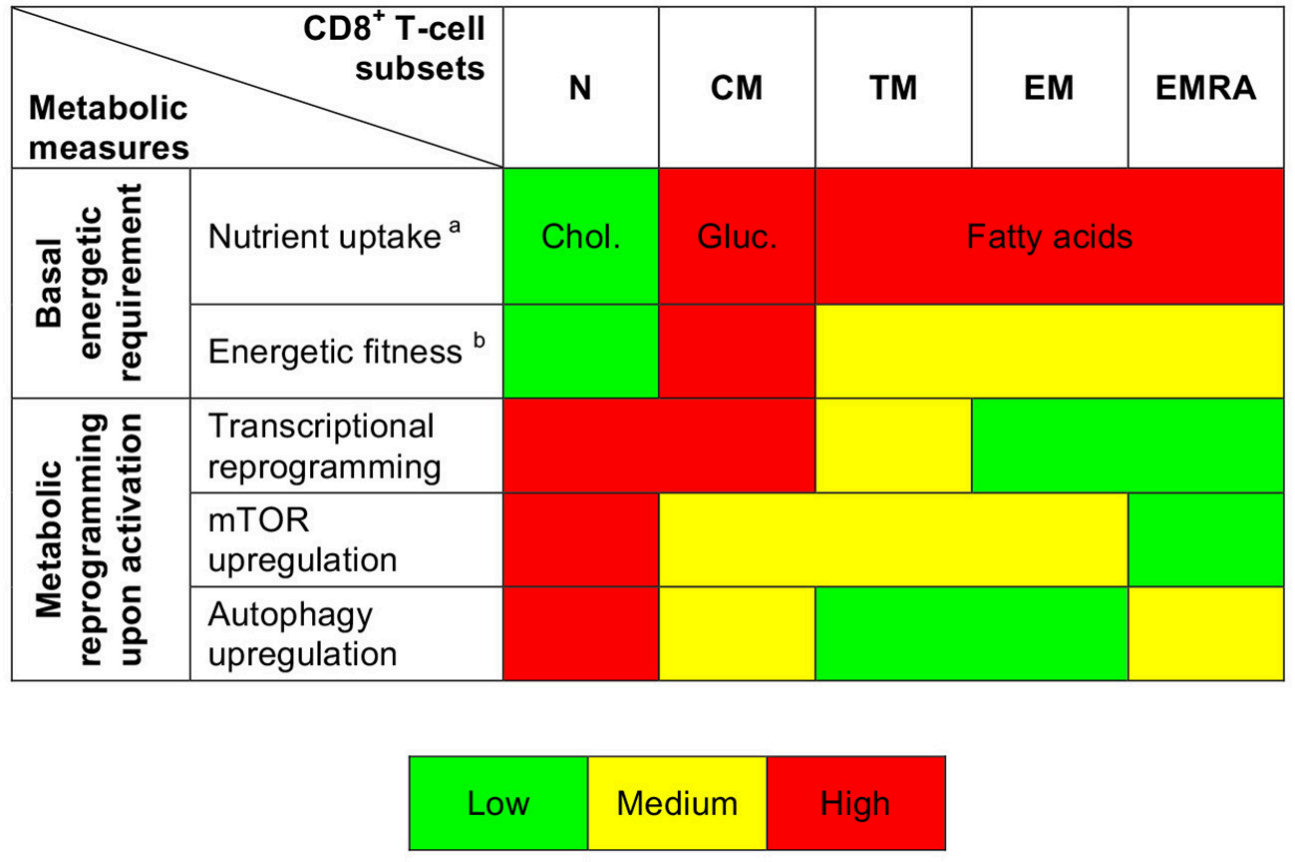
Summary depiction of the metabolic properties of CD8^+^ T-cell subsets. ^a^indicates the predominantly uptaken nutrient. ^b^is based on ROS production and mitochondrial measurements.

It has been shown previously that resting naïve and memory CD8^+^ T-cells exist in a low energy state maintained primarily via FAO (6, 18, 31–33). Our dataset confirmed and extended these observations. In particular, we found that resting naïve CD8^+^ T-cells displayed lower levels of glucose uptake, lesser mitochondrial mass, and diminished uptake of FAs compared with resting memory CD8^+^ T-cells. Moreover, we observed increased expression of glycolysis-related genes along the CD8^+^ T-cell differentiation pathway, consistent with previous reports (12, 20). Resting CM CD8^+^ T-cells exhibited higher levels of glucose uptake, greater mitochondrial mass, and lower levels of FA uptake compared with more differentiated subsets of resting memory CD8^+^ T-cells. In line with these findings, earlier work showed that CM CD8^+^ T-cells rely primarily on FAO, fueled by the conversion of glucose into FAs (31). Differentiation therefore governs the bioenergetic requirements of CD8^+^ T-cells in a subset-specific manner (Figure 6). It is tempting to speculate that such diverse energy programs reflect the contrasting homeostatic processes that regulate T-cell survival (34). For example, maintenance of the naïve CD8^+^ T-cell pool uniquely depends on tonic contacts with major histocompatibility complex class I molecules (35), and weak stimuli delivered via the TCR activate phospholipase C to generate inositol-1,4,5-trisphosphate (IP_3_) (36). Naïve CD8^+^ T-cells demonstrated relatively efficient uptake of cholesterol, which is necessary for activation of the IP_3_ receptor (37) and nanoclustering of TCRs (38).

Earlier studies have shown that T-cells undergo an mTOR-driven metabolic transition from oxidative phosphorylation to glycolysis during activation (6, 7). We found evidence of parallel roles for other metabolic pathways across the differentiation spectrum. For example, activation-induced upregulation of glycolysis-related genes and mTOR activity was most prominent among poorly differentiated CD8^+^ T-cells, which were also relatively susceptible to the inhibitory effects of 2-DG compared with EM and EMRA CD8^+^ T-cells (Figure 6). Moreover, highly differentiated CD8^+^ T-cells exhibited only moderate activation-induced upregulation of *MYC*, which is required for the classical metabolic switch (i.e., enhanced glycolysis) (22). The activation of EMRA CD8^+^ T-cells instead relied primarily on autophagy, consistent with previous observations showing that highly differentiated human T-cells downregulate components of the TCR signaling cascade and upregulate *AMPK* (AMP-activated protein kinase), which inhibits mTOR (39). The basal and activation-induced energetic profiles of human CD8^+^ T-cells therefore vary as a function of lineage and differentiation status (40).

Naïve CD8^+^ T-cells displayed a profound response to TCR-mediated activation, supported by autophagy and mTOR activity. This exceptional metabolic program may account for some of the functional qualities attributed to naïve CD8^+^ T-cells, such as the potential to generate more potent cancer-specific effector CD8^+^ T-cells (41, 42). However, it remains to be determined how this metabolic switch relates to basal quiescence, low levels of FA uptake and storage, enhanced stemness, which is associated with low mitochondrial membrane potential (43), and/or the influx of free cholesterol, which is required for T-cell activation (10).

To confirm a role for certain metabolic processes in the activation of naïve CD8^+^ T-cells, we exploited an *in vitro* priming model that recapitulates the complex interactions among immune cells *in vivo*. Using this approach, we were able to evaluate the impact of various metabolic inhibitors on the generation of antigen-experienced CD8^+^ T-cells, both in terms of magnitude and quality. However, it should be noted that we focused specifically on early priming events, potentially limiting our ability to detect metabolic processes that could affect subsequent expansion, such as lipid synthesis (44). Despite this caveat, *in vitro* priming experiments showed that the expansion and maturation of naïve CD8^+^ T-cells were strongly dependent on glycolysis, consistent with earlier observations in murine models (45). The activity of mTOR is known to support glycolysis (23). Of note, we found that rapamycin inhibited the expansion of naïve CD8^+^ T-cells, but not the acquisition of effector functions. In addition, inhibition of glycolysis had more profound effects on the expression of activation markers compared with inhibition of mTOR. These data are compatible with a role for mTOR-independent pathways in the control of lymphocyte effector functions, supported by previous observations showing that immediate-early glycolysis is mTOR-independent in CD8^+^ T-cells (8). Indeed, aerobic glycolysis may control effector functionality via epigenetic and post-transcriptional mechanisms (46, 47) and signaling intermediates (48). It is also important to recognize that mTOR is a master regulator of metabolism, such that rapamycin pre-treatment may both inhibit glycolysis and favor the upregulation of other pathways that support T-cell activation. In line with this notion, we found that autophagy was also essential for the expansion and maturation of naïve CD8^+^ T-cells. This observation contrasts with the findings of previous studies using Atg7-deficient mice, suggesting an inter-species difference in the metabolic processes that control antigen-driven responses among naïve CD8^+^ T-cells (49, 50). Our findings are nonetheless consistent with previous work in humans showing that TCR-mediated stimulation can induce both autophagy and mTOR activity in early differentiated CD8^+^ T-cells (51), and that mTOR activity is supported by the induction of autophagosomes (52). Moreover, mTOR does not always impair autophagy, which is rather p38-dependent, at least in terminally differentiated T-cell subsets (53), and our results do not exclude the possibility that mTOR activation may suppress autophagic flux at later time points (24).

Our data further revealed that FA uptake and storage in the resting state correlated inversely with mTOR activity, and that L-carnitine promoted the effector differentiation of naïve CD8^+^ T-cells. FAO may therefore favor the expression of mTOR-dependent molecules, such as the transcription factor Tbet, leading to enhanced functionality and greater immune efficacy (28–30). In line with this interpretation, high concentrations of neutral lipids and FAs, as well as inhibition of FA metabolism, have been shown to suppress proliferation, increase apoptosis, and alter mitochondrial metabolism in lymphocytes (27, 54, 55). These effects may be amenable to therapeutic manipulation, potentially enhancing suboptimal immune responses, for example in nutrient-poor tumor microenvironments (28–30).

In conclusion, we have demonstrated that substantial metabolic heterogeneity exists among phenotypically-defined subsets of human CD8^+^ T-cells. These observations hold potential biological relevance in light of previous reports showing that natural and vaccine-induced T-cells mediate protection against different pathogens as a function of differentiation (56–58) and that dysfunctional T-cells accumulate in many pathological conditions (59, 60). Metabolic regulators may therefore play a key role in novel strategies designed to correct lymphocytic anomalies and/or optimize the efficacy of vaccines and immunotherapies (61).

## Author Contributions

FN and VA: conceptualization; FN and MC-P: methodology; FN and JF: formal analysis; FN, LP, JF, and MC-P: investigation; EC, EG, AT, and DP: resources; FN and VA: writing and original draft; FN, JF, AT, DP, AC, and VA: writing, review, and editing; VA: supervision; AT, DP, AC, and VA: funding acquisition.

### Conflict of Interest Statement

The authors declare that the research was conducted in the absence of any commercial or financial relationships that could be construed as a potential conflict of interest.
