# Supplementary material for: Naïve CD8+ T-Cells Engage a Versatile Metabolic Program Upon Activation in Humans and Differ Energetically From Memory CD8+ T-Cells
Source: Front Immunol. 2018 Dec 21;9:2736. doi: 10.3389/fimmu.2018.02736 (PMC6308131; doi:10.3389/fimmu.2018.02736)
Supplement: Supplementary file 1 [file Data_Sheet_1.pdf]

Supplementary Material

Human Naïve CD8$^+$ T-Cells Show Different Energetic Requirements Compared to Memory Cells and Simultaneously Engage mTOR and Autophagy Upon Activation

Francesco Nicoli*, Laura Papagno, Justin J. Frere, Mariela Cabral-Piccin, Emmanuel Clave, Emma Gostick, Antoine Toubert, David A. Price, Antonella Caputo, Victor Appay*

***Correspondence**

Victor Appay: victor.appay@upmc.fr

Francesco Nicoli: nclfnc1@unife.it

1.1 Supplementary Figures

Figure S1. Gating strategy for CD8$^+$ T-cell subsets. CD3$^+$ events were gated as CD8$^+$. Differential expression of CD27, CD45RA, and CCR7 was used to identify subsets as naïve (N), central memory (CM), transitional memory (TM), effector memory (EM), or terminally differentiated effector memory (EMRA).

Figure S2. PD-1 expression in activated CD8$^+$ T-cell subsets. PBMCs were activated with plate-bound αCD3. Expression of PD-1 was measured by flow cytometry after 24 hr. Left panel: one representative example is shown. Right panel: horizontal lines depict mean values. N = 15. Statistical significance was determined using a one-way paired ANOVA with Bonferroni's post-test. ** $P < 0.01$, *** $P < 0.001$.

Figure S3. Antigen-specific priming of naïve CD8$^+$ T-cells depends on autophagy and mTOR. (**A–D**) MelA-specific naïve CD8$^+$ T-cells were primed in the absence or presence of various metabolic inhibitors. (**A and D**) MelA-specific CD8$^+$ T-cells were quantified by flow cytometry after 10 days using cognate PE-conjugated ELA/HLA-A2 tetramers. Expression of granzyme B (**B**) and Tbet (**C**) in primed MelA-specific CD8$^+$ T-cells was measured by flow cytometry after 10 days. Horizontal lines represent mean values. Statistical significance was determined using the Wilcoxon signed rank test. N = 7 (A-C); N = 8 (D). * $P < 0.05$.

1.2 Supplementary Tables

Table S1. Directly conjugated antibodies used for flow cytometry staining

Table S2. Gene expression in CD8$^+$ T-cell subsets.

Table S3. Basal metabolic properties of CD8$^+$ T-cell subsets.

Table S4. Response to activation of CD8$^+$ T-cell subsets.

Table S5. Metabolic switch after activation in CD8$^+$ T-cell subsets.

Table S6. Effect of metabolic inhibition on activation in CD8$^+$ T-cell subsets.
