# Supplementary material for: Naïve CD8+ T-Cells Engage a Versatile Metabolic Program Upon Activation in Humans and Differ Energetically From Memory CD8+ T-Cells
Source: Front Immunol. 2018 Dec 21;9:2736. doi: 10.3389/fimmu.2018.02736 (PMC6308131; doi:10.3389/fimmu.2018.02736)
Supplement: Supplementary file 2 [file Data_Sheet_2.pdf]

Table S1

ANTIBODY	FLUOROCHROME	SUPPLIER AND REFERENCE
**CCR7**	BV650	BD BIOSCIENCES - 563407
**CCR7**	PE-Cy7	BD BIOSCIENCES - 557648
**CD134**	BV711	BD BIOSCIENCES - 563664
**CD27**	PE	BD BIOSCIENCES - 555441
**CD27**	AF700	BIOLEGEND - 302814
**CD27**	BUV395	BD BIOSCIENCES - 563815
**CD3**	BV605	BD BIOSCIENCES - 563219
**CD38**	PC7	BECKMAN COULTER - A54189
**CD4**	APC-Cy7	BD BIOSCIENCES - 557871
**CD4**	BUV395	BD BIOSCIENCES - 563550
**CD4**	HV500	BD BIOSCIENCES - 560768
**CD40L**	PE	BD BIOSCIENCES - 555700
**CD45RA**	ECD	BECKMAN COULTER - IM2711U
**CD45RA**	PerCP-Cy5.5	EBIOSCIENCES - 45-0458-42
**CD45RA**	V450	BD BIOSCIENCES - 560362
**CD49d**	PE-Cy7	BIOLEGEND - 304314
**CD57**	Pacific Blue	BIOLEGEND - 322316
**CD69**	FITC	BD BIOSCIENCES - 347823
**CD8**	APC	BD BIOSCIENCES - 555369
**CD8**	APC-Cy7	BD BIOSCIENCES - 557834
**CD8**	FITC	BD BIOSCIENCES - 555366
**Granzyme B**	V450	BD BIOSCIENCES - 561151
**HLADR**	PE-CF594	BD BIOSCIENCES - 562304
**PD1**	APC	BIOLEGEND - 329908
**pS6**	Pacific Blue	CELL SIGNALLING - 8520S
**Tbet**	eFluor660	EBIOSCIENCES - 50-5825-80
