# Supplementary figures and images for: Naïve CD8+ T-Cells Engage a Versatile Metabolic Program Upon Activation in Humans and Differ Energetically From Memory CD8+ T-Cells
Source: Front Immunol. 2018 Dec 21;9:2736. doi: 10.3389/fimmu.2018.02736 (PMC6308131; doi:10.3389/fimmu.2018.02736)

Figure S1

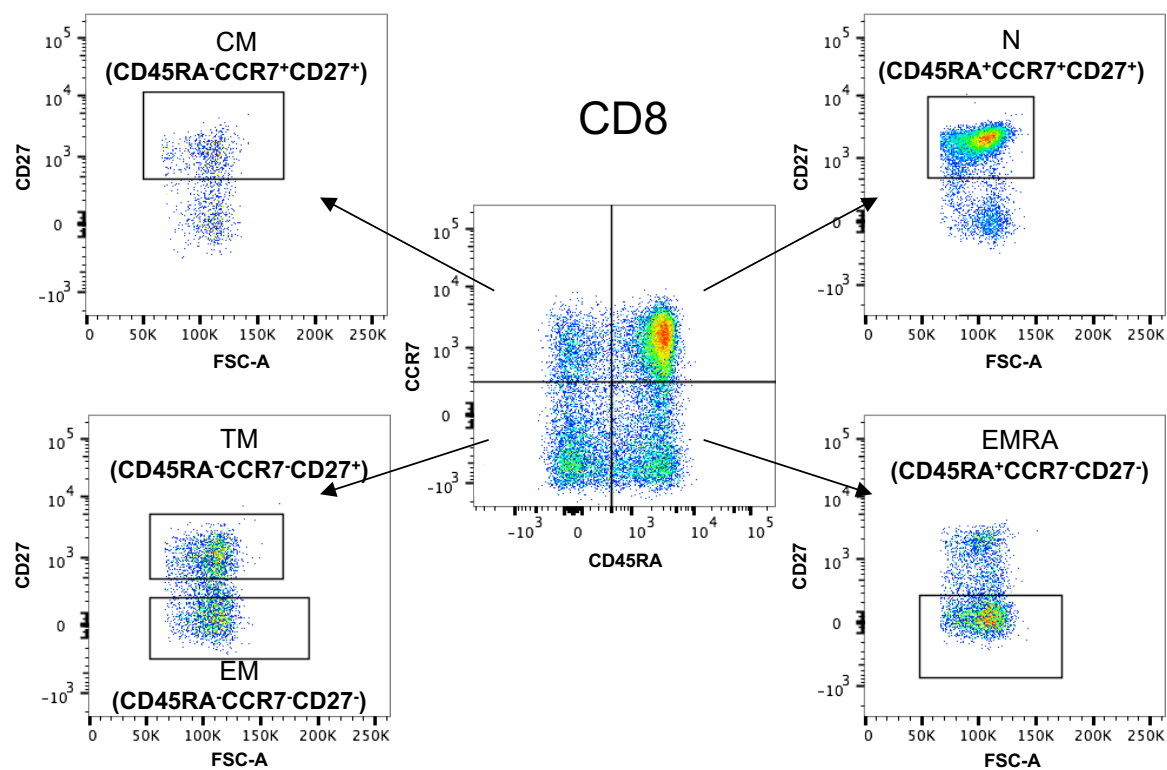

Figure S2

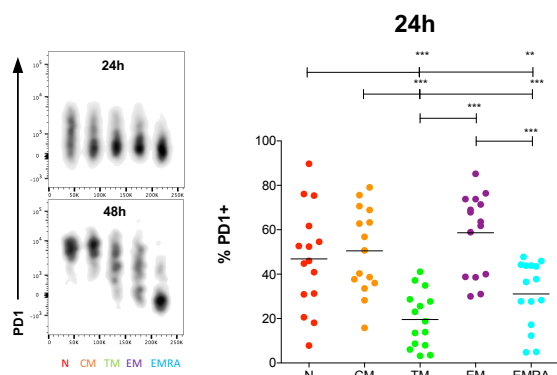

Figure S3

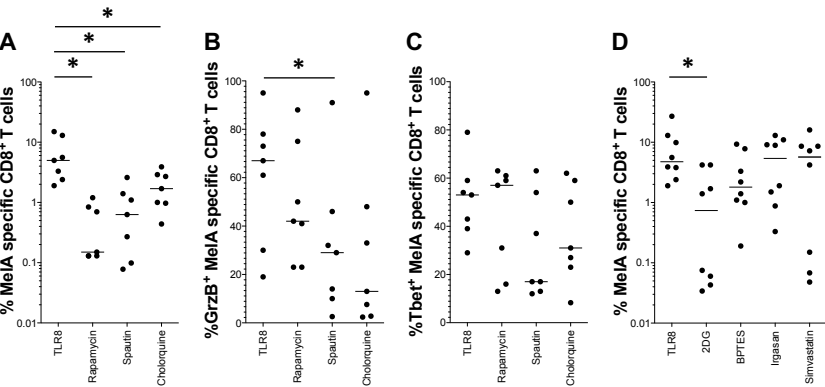

Supplement: Supplementary file 3 [file Data_Sheet_3.pdf]
